# Feasibility of the Minimally Invasive Lumbar Decompression Procedure in a Lumbar Stenosis Patient With Radiographic Evidence of Spinal Instability

**DOI:** 10.7759/cureus.86825

**Published:** 2025-06-26

**Authors:** Ahmed Elnahla, Karam Asmaro, Adnan Hussain

**Affiliations:** 1 Anesthesiology, Perioperative Medicine and Pain Management, Henry Ford Health, Detroit, USA; 2 Neurosurgery, Henry Ford Health, Detroit, USA

**Keywords:** lumbar spinal stenosis (lss), mild procedure, minimally invasive lumbar decompression, neurogenic claudication, spinal instability

## Abstract

Lumbar spinal stenosis (LSS) can be challenging to treat in certain patient populations, particularly in patients for whom medical management is ineffective and surgical interventions carry a high risk of complications. This case report describes an 83-year-old woman with rheumatoid arthritis and LSS who presented with neurogenic claudication. Diagnosis was confirmed by physical examination and imaging, revealing canal stenosis and lumbar instability. Conservative measures failed to improve her symptoms, and she was deemed a poor surgical candidate given her age, advanced arthritis, and her current immunotherapy. Despite lacking supporting evidence, spinal instability has been considered a contraindication for minimally invasive lumbar decompression (MILD). However, following a multidisciplinary discussion, MILD was offered to the patient as a treatment option. To our knowledge, this is the first case of MILD in a patient with lumbar instability, resulting in sustained pain relief lasting over a year.

## Introduction

Lumbar spinal stenosis (LSS) is a degenerative spine disorder characterized by substantial pain and limitations in functional capabilities [1]. It affects approximately 11% of the older adult population in the United States. With a larger aging population, the prevalence of LSS is anticipated to rise. Patients typically present with back pain, lower extremity pain, numbness, and, occasionally, weakness [2]. These symptoms are disabling, often requiring intermittent resting, hence the name neurogenic claudication. Despite its high prevalence, the majority of patients with LSS remain asymptomatic, with around 10% developing symptoms by the age of 70 years [3].

LSS is defined as a condition in which the space available for the neural and vascular structures in the lumbar spine is diminished secondary to degenerative changes in the spinal canal. This narrowing can be attributed to either anterior or posterior pathology. The anterior is essentially due to disc degeneration, and the posterior is either due to posterior ligamentum flavum hypertrophy (LFH) or facet arthropathy [1].

Treatment is often challenging, necessitating the use of various treatment approaches. Typically, initial treatment comprises medical and physical therapy. Interventional options such as epidural steroid injections are frequently offered. However, while these interventions may offer some relief, they often fail to deliver durable and lasting results. Additionally, spinal cord stimulation has been tried; however, there is no strong evidence supporting its use in patients with LSS, likely due to the mechanical nature of the problem. Decompression surgery, minimally invasive or open surgery, stands as the golden standard and definitive treatment, but may not be an ideal option for patients with advanced medical comorbidities [4].

Minimally invasive lumbar decompression (MILD) is a relatively recent procedure for treating LSS. It involves decompressing the spinal canal space by removing a small portion of the lamina and ligamentum flavum [5]. Initially developed in 2005 by Dr. David Solsberg and Dr. Donald Schomer as a therapeutic approach for poor surgical candidates with spinal stenosis. The FDA granted approval in 2006, after which the first patient underwent treatment promptly post-approval. Over the ensuing years, multiple studies have validated the efficacy of the MILD procedure. In 2016, the MiDAS ENCORE trial reported superior statistical outcomes of MILD over epidural steroid injections in treating selected LSS patients. This led to Medicare coverage approval for MILD in 2017 [5,6].

Ideally, candidates for MILD should meet certain criteria after unsuccessful conservative treatment, including patients with symptomatic central LSS and radiographic evidence of spinal stenosis (LFH ≥ 2.5 mm and effacement of the cerebrospinal fluid space within the theca sac). It is important to emphasize that spinal instability, evaluated primarily through imaging such as flexion-extension X-rays, often requires the addition of stabilization and fusion to avoid worsening symptoms [6].

In our patient, an individualized risk-benefit assessment led to the decision of offering the MILD procedure, which resulted in a favorable post-procedure outcome. To our knowledge, this is the first case of MILD procedure in an LSS patient with radiographic evidence of spinal instability.

## Case presentation

An 83-year-old female with a medical history of rheumatoid arthritis and LSS was referred to our pain clinic by her orthopedic surgeon in May 2023. She presented with worsening back pain and bilateral leg pain, more pronounced on the left side, persisting for over two months. Her pain exhibited characteristics consistent with neurogenic claudication, including a positive shopping cart sign. Her functional mobility and walking distance progressively declined due to the persisting pain. Her physical examination was notable for lumbar paraspinal tenderness, positive facet loading, and a positive straight leg test on the left.

**Figure 1:**
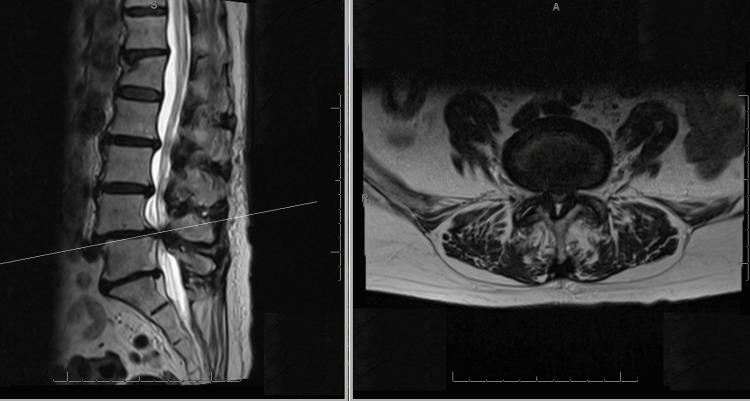
T2-weighted sagittal (left) and axial (right) MRI of the lumbosacral spine showing diffuse degenerative disc disease. There is marked central canal stenosis at the L4-5 level due to ligamentum flavum hypertrophy, with ligamentum flavum measuring 3.9 mm.

MRI findings revealed diffuse degenerative disease with moderate central canal stenosis at L2-4 and severe stenosis at L4-5. This was primarily attributed to LFH measuring 3.9 mm, along with disc bulging and hypertrophic facet arthropathy (Figure 1). Additionally, there was moderate to significant bilateral foraminal narrowing secondary to grade 1 anterolisthesis.

**Figure 2:**
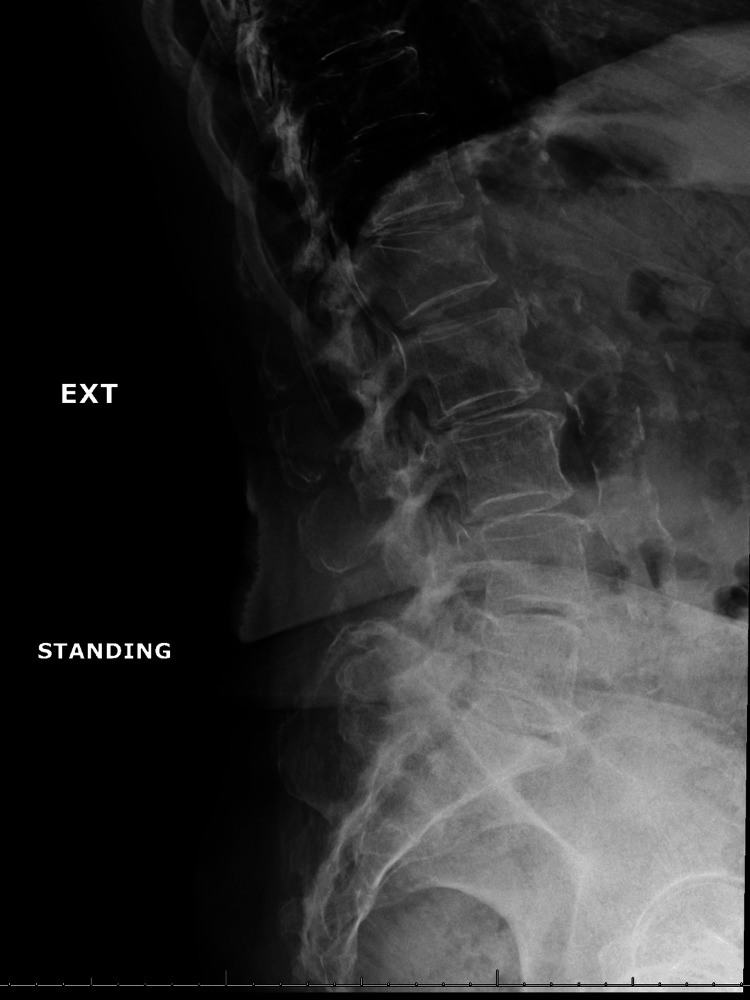
Lumbosacral X-ray extension view showing grade 1 retrolisthesis of L1 on L2 and L2 on L3.

**Figure 3:**
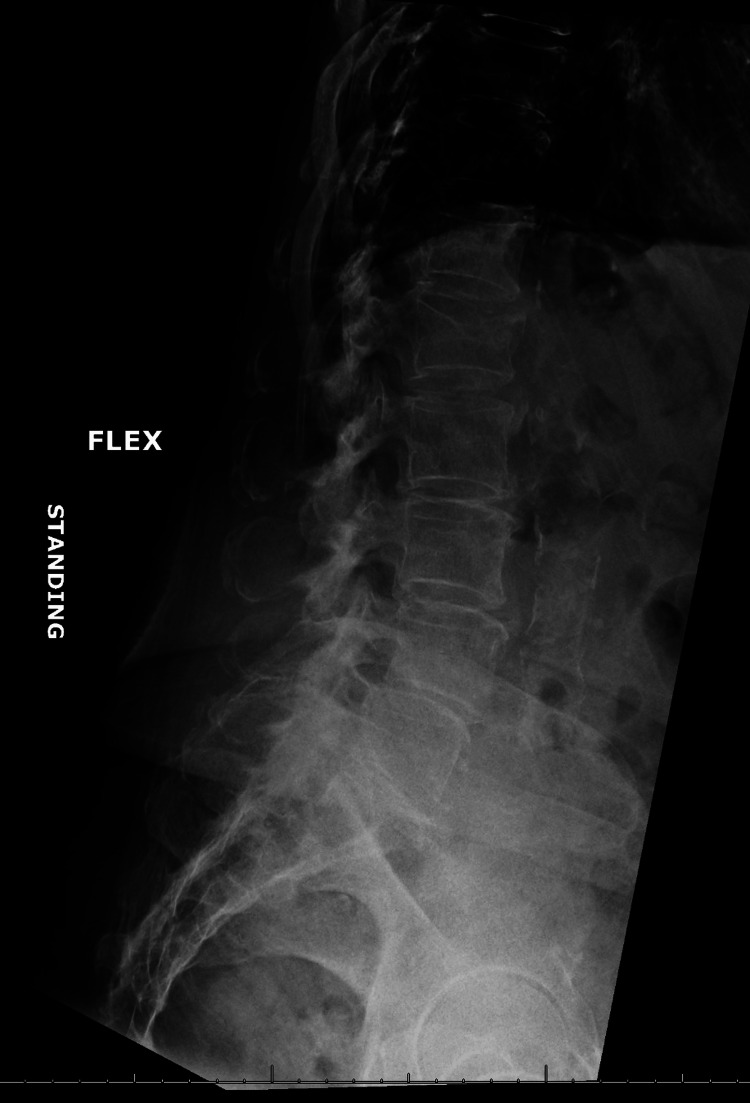
Lumbosacral X-ray flexion view showing improvement of retrolisthesis of L1 on L2 and L2 on L3.

We elected to manage her pain with L5-S1 interlaminar lumbar epidural steroid injection; however, pain relief was limited to less than 50% and only lasted for a few days. Two months later, she underwent left L5 and left S1 transforaminal epidural steroid injection; however, again, there was no significant improvement in her pain level or function. Further imaging evaluation was notable for spinal instability on her flexion-extension lumbar X-rays, evidenced by grade 1 retrolisthesis of L1 on L2 and L2 on L3 that improves on flexion (Figures 2, 3).

Given the limited efficacy of the epidural injections, we considered the MILD procedure as a treatment option to alleviate lumbar spine compression. However, the potential risk of exacerbating spine instability, particularly in the context of dynamic instability, prompted a comprehensive discussion with the neurosurgery team. Given the heightened risk of infection associated with the patient’s current medical treatment for rheumatoid arthritis and risk for pseudoarthrosis with instrumentation, given the advanced age and chronic rheumatoid arthritis treatment, the neurosurgery team recommended proceeding with the MILD procedure, with the option for surgery if the outcomes proved to be limited. The patient actively participated in this deliberation and ultimately chose to undergo the MILD procedure.

The patient underwent the MILD procedure at the L4-5 and L3-4 in October 2023, resulting in significant improvement in both pain and functional status. Pain intensity, as assessed by the Visual Analog Scale, improved markedly from a consistent baseline range of 6-8 to 0-2 on follow-up visits. In parallel, her Patient-Reported Outcomes Measurement Information System Pain Interference T-scores decreased from 73 and 72 pre-procedure to 55 and 57 post-procedure, reflecting a substantial reduction in pain-related functional limitations. Clinically, she reported the ability to ambulate significantly longer distances and increased participation in household physical activities. Follow-up over a 12-month period demonstrated sustained improvement and a favorable long-term outcome.

## Discussion

The treatment of LSS has evolved over the years, ranging from conservative management with medical and physical therapy to open spine surgery, including laminectomy with or without fusion. Typically, surgical intervention is reserved for patients who fail conservative management or with advanced disease who are unlikely to benefit from conservative management. However, surgical treatment, while providing a definitive solution for LSS, carries the inherent risks of general anesthesia due to its open and invasive nature. Minimally invasive approaches have mitigated the invasive nature of open surgery. However, it may not always result in complete resolution of symptoms, rendering its efficacy variable. Additionally, patients undergoing surgery are also susceptible to chronic post-surgical pain, including conditions such as post-laminectomy syndrome [7,8].

MILD is a treatment option when conservative measures fail to address the pain, and yet the patient is a poor surgical candidate for spine surgery. It is a relatively recent intervention, which was initially designed to treat cancer patients who were deemed poor candidates for surgical interventions. Shortly after being approved, several studies were conducted demonstrating its efficacy with promising results. Later, in 2016, after the Benyamin et al. clinical trial, the US Centers for Medicare and Medicaid Services approved nationwide Medicare coverage of MILD in 2017 [1,5].

Previous studies on MILD have shown its superior effectiveness compared to epidural steroid injections, providing long-term benefits by reducing pain and improving function [9]. Furthermore, based on these promising results, some studies have suggested that the MILD procedure should be considered a first-line intervention for selected patients [10].

While MILD does not involve the removal of a significant portion of the ligamentum flavum, as in surgical decompression, its efficacy in achieving substantial pain relief can be elucidated. Reports suggest that even a slight increase in LFH may contribute to significant pain. Conversely, the removal of small amounts of LFH through MILD can offer noteworthy relief [1]. Similarly, previous studies have shown that a minor reduction in disc volume could lead to a substantial change in intradiscal pressure, thereby alleviating discogenic low back pain. Altogether, LSS is nuanced and can be due to multiple factors. While MILD can decompress the theca sac from LFH, it does not address facet arthropathy and hypertrophy as it often impinges the theca sac. Due to the multifaceted nature of the disease process, a multidisciplinary evaluation is recommended to choose the best treatment option for the patient.

As of the present moment, there is a notable absence of studies directly comparing the efficacy, safety, or complication rates between MILD and decompressive procedures or surgeries. Nonetheless, existing literature suggests that MILD demonstrates a notably safe profile, with the lowest reported complications, similar to an epidural steroid injection. In general, patients do not require a hospital admission and can resume their normal activities sooner after the procedure compared to open surgery [8,10,11].

A recent study spanning five years reported about 12% failure rate for MILD, necessitating surgical decompression. Interestingly, upon closer examination, only two patients reported post-surgery improvement, suggesting potentially inadequate decompression during the initial MILD procedure or compression due to other etiologies rather than LFH alone. One patient experienced worsening symptoms, three reported no change, and three were lost to follow-up. While the available data may not suffice for definitive conclusions, prolonged pain duration and specific pain characteristics could be contributory factors [8].

It is worth noting that during MILD, only a small part of the bony tissue is removed. The surrounding ligaments, the lamina, and the facet joints remain intact. Therefore, some studies reported that it does not affect spinal stability. This can be somewhat supported by the absence of post-MILD or MILD-induced instability; however, it has to be taken into consideration that these reports did not have spinal instability to begin with. Of note, since being approved, the FDA has not stated a contraindication for its use in spinal instability [7]. Post-procedure flexion and extension X-rays did not show worsening instability in our patient.

In our patient’s case, the dilemma centered around offering the MILD procedure, as there were concerns that it may worsen her spinal instability. In theory, the removal of a part of the lamina and the ligamentum flavum is expected to compromise stability in a patient with evidence of spinal instability, despite limited support from the existing literature. Our patient did have evidence of spinal instability on her flexion-extension X-rays (Figures 2, 3). Given the elevated risk of infection associated with the patient’s current medical treatment for rheumatoid arthritis, a prompt discussion with our neurosurgery colleagues who recommended proceeding with the MILD procedure and reserving surgical intervention as a secondary alternative. Our case report is limited by the relatively short-term follow-up, and further close attention is necessary to ensure no worsening instability and pain in the future.

## Conclusions

The MILD procedure represents a safe and effective treatment option for patients with central LSS. As with most procedures, patient selection is critical to success. In general, these patients should demonstrate clinically significant symptomatic neurogenic claudication and have imaging evidence of LFH. However, the literature shows variability regarding certain aspects of the procedure, such as LFH thickness and the degree of spondylolisthesis. Therefore, the decision to offer MILD should be carefully evaluated on a case-by-case basis. To our knowledge, this is the first report of the MILD procedure being performed in a patient with LSS and radiographic evidence of dynamic spinal instability, resulting in a sustained favorable outcome over a one-year follow-up period.
